# Profiling of Nutritional and Health-Related Compounds in Oat Varieties

**DOI:** 10.3390/foods5010002

**Published:** 2015-12-25

**Authors:** Hetty C. van den Broeck, Diana M. Londono, Ruud Timmer, Marinus J. M. Smulders, Ludovicus J. W. J. Gilissen, Ingrid M. van der Meer

**Affiliations:** 1 Wageningen University & Research Centre, Plant Research International, P.O. Box 16, 6700 AA Wageningen, The Netherlands; dianalondono7@hotmail.com (D.M.L.); rene.smulders@wur.nl (M.J.M.S.); luud.gilissen@wur.nl (L.J.W.J.G.); ingrid.vandermeer@wur.nl (I.M.M.); 2 Wageningen University & Research Centre, Applied Plant Research, P.O. Box 430, 8200 AK Lelystad, The Netherlands; ruud.timmer@wur.nl

**Keywords:** antioxidants, β-glucan, fatty acids, human health, oats, tocotrienol, vitamin E

## Abstract

The use of oats in the human diet has decreased over the past 70 years. This is an unfortunate development from the perspective of human health because oats have a high nutritional value and contain many compounds, including β-glucan, polyphenols, vitamins, and unsaturated fatty acids that are able to maintain or may even improve consumer’s health. In addition, oats fit into a gluten-free diet of celiac disease patients because they lack the T-cell stimulating epitopes from wheat, rye, and barley. We focused on the presence of health-related compounds in oats and how their levels vary among varieties in response to the type of soil. Ten oat varieties were grown in the Netherlands in sandy and clay soil and were analyzed for the presence and concentration of healthy compounds (β-glucan, fatty acids, vitamin E, and antioxidant activity), avenin composition, total protein and starch content, and agronomical characteristics. Principal component analysis showed that genetic background influenced the levels of all analyzed components. Protein, starch, β-glucan, and antioxidants were also affected by the type of soil. The obtained results showed that this kind of analysis can be used to profile oat varieties in general and enables the selection of specific varieties with specific compound characteristics.

## 1. Introduction

After a world-wide decline over the last 70 years, interest in and production of oats is growing again, especially because of increased awareness of its health-related characteristics in food and feed, and because oats have sustainability characteristics including low-input need, high disease resistance, and soil-improving properties in crop rotations [1]. Oat is increasingly recognized as a healthy cereal with eminent protein composition and is accepted as safe for celiac disease (CD) patients who are intolerant to gluten proteins from wheat, rye, and barley [2,3]. It was shown that oat prolamin proteins, called avenins, do not contain the CD immunogenic epitopes as the gluten proteins from wheat, rye, and barley that induce CD [4]. The main storage proteins in oats are globulins, in contrast to the main storage proteins in wheat, rye, and barley, being prolamins [5].

Oats are the cereals having the highest content of soluble fibers, which are β-glucans [6,7]. β-Glucans can help in lowering blood cholesterol (LDL) as was shown in several clinical studies [8,9,10]. The health claim of oat β-glucans on cholesterol-lowering activity has been approved by the U.S. Food and Drug Administration [11] and by the European Food Safety Authority (EFSA) [12,13]. Aside from its cholesterol-lowering potential, β-glucan can stimulate the immune system and positively affects the functioning of the human intestinal flora [14,15]. The average total dietary fiber content in oats is 10.3%, of which 3.8% is soluble [16]. In contrast to wheat in which the average total dietary fiber content is 12.6% of which only 2.3% is soluble fiber [16]. The starch in oats is slowly but completely degradable with a balancing effect on postprandial blood glucose levels, which is useful in patients having diabetes. This is again because of the high levels of β-glucan being present [14,17].

Oats also have the highest fatty acid content among all cereals. They are a good source of linoleic acid and contain low amounts of saturated fat [15,18,19], which can help reduce the risk of heart and vascular diseases [20]. Unsaturated fatty acids (UFAs) are considered to be healthy compounds and oats contain the highest levels of UFAs compared with all other cereals. The main fatty acids present in oats are mono-unsaturated fatty acid (MUFA, C18:1) and poly-unsaturated fatty acid (PUFA, C18:2) [18,19], followed by saturated fatty acid (C16:0). The fat-soluble antioxidants (vitamin E) present in oats prevent the fatty acids from becoming oxidized by lipases and lipoxygenases. When oats are used in food products, inactivation of lipases and lipoxygenases performed by steaming, kilning, or roasting, is essential to prevent off-flavor in oat flour and broken or damaged oat grains [21]. High intake of vitamin E via food, of which α-tocotrienol and α-tocopherol have been reported to be mainly present in oats, may contribute to the prevention of age-dependent neurodegenerative diseases such as Alzheimer’s disease [22].

Unique phenolic antioxidants present in oats are the avenanthramides [15,23,24], which have shown anti-inflammatory activity *in vitro* [25]. The antioxidant components have the capacity to prevent the oxidation of LDL cholesterol and have a potential in skin care [26,27]. Oats are mostly consumed as a whole grain; for this reason, the antioxidant components present in the germ and bran, such as avenanthramides, are included. Furthermore, oat germ is rich in vitamins and minerals.

The presence of many health-promoting compounds makes oats interesting cereals to be used in food and bakery products. Presently, oats are increasingly becoming advertised as a “superfood” (Cereal Partners Worldwide, 2013). This increasing interest in oats has potential for breeding, cultivation, product design, and product marketing. Crop and product improvement can be served by better understanding of metabolic and phenotypic characteristics and correlations among them. In a first approach for that, we have chosen to study the following variables in flour of ten oat varieties grown in two different soil types (clay and sand): (1) End products of primary metabolism (*i.e.*, protein contents, including the avenin component; starch, including starch damage as a measure for industrial food quality, especially water absorption; individual fatty acids, and β-glucans); (2) End products of secondary metabolism (*i.e.*, vitamin E-related phenolics and total anti-oxidant activity); (3) Field characteristics (*i.e.*, lodging, yield, and thousand kernel weight). The data of these specific characteristics have been analyzed by principal component analysis (PCA) to identify correlations among variables and correlations with variety, soil type, or both. The underlying data from individual varieties grown in different soils have been identified. The objective of this study was to analyze the biological variation between ten oat varieties grown in the Netherlands in clay soil and sandy soil regarding primary metabolites, secondary metabolites, and agronomic data. The implications and potentials of this approach for oat breeding and crop cultivation, as well as the potential for specific product applications are discussed.

## 2. Experimental Section

### 2.1. Field Trial

Field trials were performed with the following ten *Avena sativa* spring varieties: Ascot (Swe), Astor (NL), Gambo (NL), Gele van Timmermans (NL), Gigant (NL), Leanda (NL), Mansholt III (NL), Panache de Roy (FR), Troshaver uit Besel (NL), and Zandster (NL). They were set up in two soil types in the Netherlands: clay soil (Lelystad, The Netherlands) and sandy soil (Rolde, The Netherlands). At each location the oat varieties were planted in triplicate on 3 × 24 m plots, at a density of 250 seeds per m^2^. Sowing was done mechanically and cultivation was according to Good Agricultural Practice (GAP). Nitrogen was applied according to the plants’ need and depending on the soil. During cultivation, plants were monitored for their resistance to lodging. Four months after planting, ripe seeds were harvested per plot. Yield (kg/ha at 15% moisture) was calculated and the value for thousand kernel weight (TKW at 15% moisture) was determined.

### 2.2. Kilning and Milling of Oat Seeds

The seeds of the individual plots were pooled per variety and per soil type, de-hulled (at De Halm, Heeswijk-Dinther, The Netherlands), and kilned to inactivate lipase activity. During de-hulling only the outer husks were removed and afterwards the bran and aleurone were still present. The kilning process was performed by steam heating at 100 °C for 3 min on seeds in 3 cm-thick layers in a 0.5 m^3^ steel container, followed by overnight cooling down to room temperature in a drying oven, starting at 85 °C. Lipase inactivation by the kilning process was checked in representative samples by measuring peroxidase activity as indicator [28] with unkilned material as control. The kilned material was milled using a laboratory scale hammer mill (MAGICO EM50).

The obtained oat flours were analyzed for moisture content, amount of total protein, total starch, damaged starch, fatty acids, vitamin E, and β-glucan. Furthermore, antioxidant capacity and avenin profiles were analyzed.

### 2.3. Starch, Total Protein, and Moisture Content

Total starch and damaged starch contents were quantified in duplicate, according to AACC Approved methods 76-13 and 76-31, respectively, using Megazyme Starch and Starch damage kits [29], according to manufacturer’s instructions. Total protein content of the oat flours was determined by measuring nitrogen content with the Dumas method [30] with a NA 210 nitrogen and protein analyzer (ThermoQuest, Ronado, Italy) and the relationship N × 6.26 for oat [31], according to AACC Approved method 46-30 [29]. Moisture content was quantified in duplicate according to Approved method 44-15A [29]. All results from compound analyses were corrected for the differences in flour moisture content to a flour moisture content of 10%.

### 2.4. Avenins

From each oat flour sample, 100 mg was extracted twice with 0.5 mL of 50% (*v*/*v*) aqueous iso-propanol by mixing for 30 min at room temperature, followed by centrifugation at 10,000 rpm for 10 min. The two obtained supernatants were combined and considered the avenin protein extract [32]. The avenin protein content was quantified using the Biorad Protein Assay (Bio-Rad Laboratories, Hercules, CA, USA) in duplicate, based on the Bradford dye-binding procedure, according to manufacturer’s instructions, with BSA as standard. Oat avenins were separated on SDS-PAGE gels of 11% as described [33], using a Hoefer SE 260 mighty small II system (GE Healthcare, Little Chalfont, UK), followed by staining with PageBlue™ (Thermo Scientific, Waltham, MA, USA).

### 2.5. β-Glucans

β-Glucan concentrations in the oat flour samples was quantified in duplicate by AACC Approved method 32-23 [29], using the mixed β-glucan linkage kit from Megazyme (Bray, Ireland), according to manufacturer’s instructions.

### 2.6. Fatty Acids

Fatty acids were extracted as described by Folch *et al.* [34] but optimized for our tissue type. First, 1 mL of a chloroform:methanol mixture (2:1; *v*/*v*) containing 0.1% (*w*/*v*) butylated hydroxytoluene (BHT) was added to 25 mg oat flour. After mixing, tubes were kept on ice for 10 min. Next, 0.2 mL of a 0.6% NaCl (*w*/*v*) solution was added. The samples were mixed and kept on ice for another 10 min. Tubes were centrifuged for 10 min at 2500 rpm to separate the polar and a-polar phase. The chloroform phase was transferred to a fresh tube and the polar phase and debris were re-extracted twice with 1 mL chloroform. The chloroform fractions were pooled and dried by evaporation under a stream of nitrogen gas. The fatty acids of the dried chloroform extracts were converted into fatty acid methyl esters (FAMEs) using a 3 mL solution of 5% H_2_SO_4_ in methanol (*v*/*v*). Tubes were mixed for 15 sec and incubated at 70 °C for 3 h. Tubes were cooled to room temperature and 3 mL hexane and 3 mL milliQ water were added. Tubes were mixed for 20 s followed by centrifugation for 5 min at 2500 rpm. The hexane top phase was transferred to a Na_2_SO_4_ column to dehydrate the sample. The FAMEs were analyzed by GC–MS as described by Bouwmeester *et al.* [35]. A ZB-1 column (Phenomenex, Toorance, CA, USA, 30 m × 0.25 mm internal diameter and 0.25 μm film thickness) was used, and the column oven was programmed at an initial temperature of 45 °C for 1 min, followed by a ramp of 10 °C·min^−1^ to 310 °C and a final step of 7.5 min at 310 °C. The FAMEs were identified and quantified by means of authentic standards of C16-C22 fatty acid methyl esters mixture (Sigma-Aldrich, St Louis, MO, USA). Only main peaks were used for quantification.

### 2.7. Vitamin E

Tocochromanols were extracted as described previously by López-Raéz *et al.* [31] with some modifications. Briefly, 50 mg of oat flour was extracted with 2 mL of a methanol:chloroform mixture (2.5:2) containing 0.1% butylated hydroxytoluene (BHT). After mixing and incubation on ice in the dark for 10 min, 2.5 mL of 50 mM Tris-HCl (pH 7.5) containing 1 M NaCl added. Tubes were incubated on ice for another 10 min, followed by centrifugation for 10 min at 2500 rpm. The chloroform phase was transferred to a clean tube. Samples were re-extracted twice with 1.0 mL of chloroform containing 0.1% BHT. Chloroform fractions were pooled and dried by evaporation under a stream of nitrogen gas. The dried tocochromanols were dissolved in 1 mL ethyl acetate containing 0.1% BHT by sonication. Tocochromanols were analyzed by HPLC analysis according to Bino *et al.* [32] using a YMC-Pack reverse-phase C_30_ column (250 × 4.6 mm; 5 μm) coupled to a 20 × 4.6 mm C_30_ guard (YMC Inc., Wilmington, NC, USA), maintained at 40 °C. The mobile phase used was methanol, tert-methyl butyl ether and water:methanol (20:80, *v*/*v*) containing 0.2% ammonium acetate. Flow rate of 1 mL/min was used. Chromatography was carried out on a Waters system consisting of a No. 600 quaternary pump, No. 996 photo diode array detector (PDA) and No. 2475 fluorescence detector (FD). Data were collected and analyzed using the Waters Empower software supplied. Tocochromanols were detected by FD at excitation and emission wavelengths of 296 and 405 nm. Quantitative determination of compounds was conducted by comparison with dose-response curves constructed from authentic standards (α- and δ-tocopherol (Sigma-Aldrich, St Louis, MO, USA); α-, γ-, δ-tocotrienol (Bio-Connect, Huissen, The Netherlands)). Only main peaks were used for quantification.

### 2.8. Antioxidant Capacity

Antioxidants were extracted by addition of 1 mL 50% aqueous ethanol to 50 mg oat flour. After sonication for 15 min, tubes were centrifuged for 15 min at 2500 rpm. Antioxidant capacity was measured according to Re *et al.* [36], modified for 96-well plate analyses. Briefly, 10 µL extract was mixed with 90 µL of a 2-mm ABTS^•+^-solution (ABTS: 2,2′-Azinobis-(3-ethylbenzothiazoline-6-sulphonic acid)) in 50% aqueous ethanol, generated by adding K_2_S_2_O_8_. Rates of decrease in ABTS^•+^-radicals were measured as absorptions at 1 min intervals for 10 min at 415 nm. Antioxidant capacity of extracts was expressed as Trolox-equivalents by comparison with sequentially-diluted Trolox (0.25–1.25 mM) in 50% ethanol/0.1% citric acid. Samples were measured in triplicate.

### 2.9. Data Analysis

All data were analyzed by principal component analysis (PCA) using median normalized data in GeneMaths XT performed on logarithmic (log_2_) data. Cluster analysis was performed, and for cluster analysis, compounds and varieties were grouped together based on similarity. For the heatmap in cluster analysis, values were standardized to a median of 0. The red and green colors show values that were lower or higher than 0, respectively. Correlation coefficients have been calculated using Microsoft Excel.

## 3. Results and Discussion

The objective of this study was to analyze the biological variation between ten oat varieties grown in the Netherlands in clay soil and sandy soil regarding primary metabolites, secondary metabolites, and agronomic data. The primary metabolites analyzed included starch, starch damage (as food quality trait), protein (including avenins), fatty acids, and β-glucan. The secondary metabolites included tocopherols, tocotrienols and anti-oxidant activity (TEAC). Growth characteristics included yield, thousand kernel weight (TKW), and resistance to lodging. Obtained data are shown in Table 1 and Supplementary Table S1.

**Table 1:** Values for health-related nutritional compounds and field characteristics of ten oat varieties grown in sand and clay soil.

	Sand			Clay		
	Range	Average	Stdev	Range	Average	Stdev
Total protein (%)	10.9–16.6	13.2	1.8	11.6–15.5	13.7	1.2
Avenin content (%)	0.4–0.6	0.5	0.1	0.6–0.7	0.7	0.1
Starch content (%)	52.4–66.5	58.0	5.2	45.6–52.2	48.1	2.2
Starch damage (%)	1.8–4.0	2.7	0.7	1.4–2.2	1.7	0.3
β-Glucan (%)	3.3–4.9	4.0	0.5	3.8–5.6	4.6	0.5
C16:0 (mg/100 g)	193.5–292.9	245.1	31.8	178.7–262.3	237.0	42.8
C18:0 (mg/100 g)	11.5–33.3	20.1	7.6	10.0–29.9	16.2	5.7
C18:1 (mg/100 g)	385.0–718.1	515.4	113.7	310.5–723.3	438.5	125.9
C18:2 (mg/100 g)	532.3–748.9	626.9	69.5	500.3–849.0	608.0	104.0
C18:3 (mg/100 g)	12.3–16.1	15.1	1.2	13.3–17.1	15.2	1.2
Total FA (%)	1.2–1.8	1.4	0.2	1.0–2.0	1.3	0.3
TEAC (µmol/100 g)	190.6–356.5	305.0	46.5	373.0–478.6	415.3	40.0
α-Tocotrienol (mg/100 g)	2.8–4.4	3.4	0.5	3.0–4.4	3.7	0.4
α-Tocopherol (mg/100 g)	0.7–1.6	1.3	0.3	0.8–1.8	1.4	0.3
Yield (kg/ha)	4698–7401	6018	1194	4880–7570	6139	895
TKW (g/1000 kernels)	23.0–35.0	29.7	4.2	26.0–33.0	29.3	2.5
Lodging	6–9	8	1	4–9	7	2

**Figure 1:**
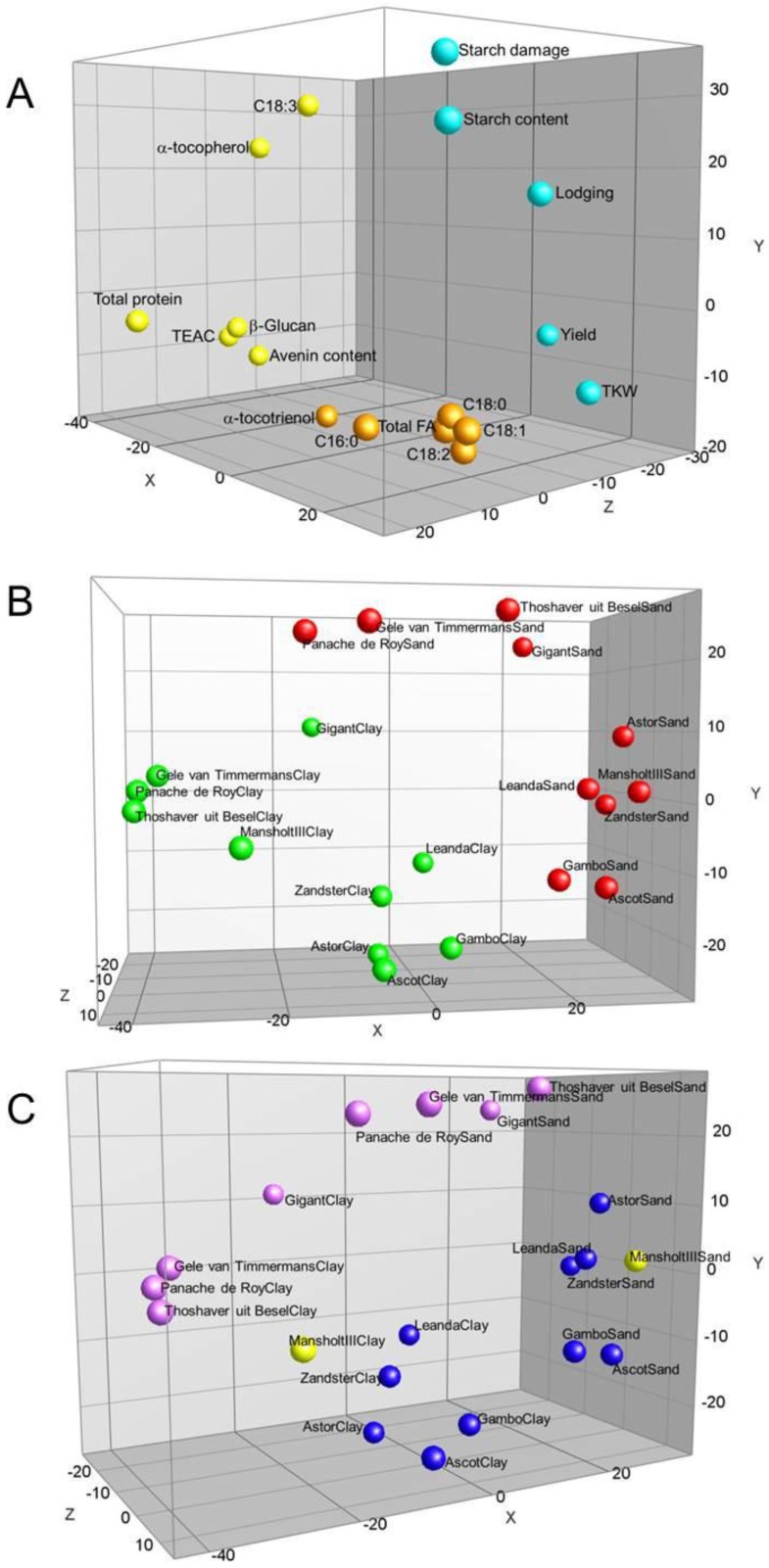
Principal component analysis 3D-plots of oat varieties and compounds. (**A**) Compound loading plot. Colors identify groups obtained from cluster analysis (Supplementary Figure S1); (**B**) Separation of oat varieties grown in sand and clay. Red dots are sand samples, green dots are clay samples; (**C**) The same as in (B), only slightly differently rotated and shows the separation of subsets of oat varieties showing contrasting compound contents when grown in either clay or sandy soil. PC1, *X*-axis, 32.0%; PC2, *Y*-axis, 21.1%; PC3, *Z*-axis, 14.3%.

Data from Table S1 were analyzed for correlation using principal component analysis (PCA). In the resulting PCA plot, the first principal component (*X*-axis) explained 32.0% of the variation, the second principal component (*Y*-axis) explained 21.1%, and the third principal component (*Z*-axis) explained 14.3% (Figure 1).

The principal components (PCs) discriminated between clay and sand grown samples (Figure 1B) and divided the varieties in different subsets that show contrasting effects for the different compound levels (Figure 1C). The levels of compounds C18:3 and α-tocopherol were higher in a subset of varieties irrespective of the type of soil. The levels of all compounds were influenced by the variety and were thus determined by the genetic background. Some compounds were also affected by growing in either sandy or clay soil independent of the genetic background of the oat variety, while others were negatively influenced by sand or clay in all varieties. These results show that the choice of soil for cultivation can affect the compound profile of oat kernels. The starch content on the one hand, and protein and β-glucan contents on the other hand, showed an inverse relationship: starch content (and related starch damage) was relatively high in all varieties when grown in sand; β-glucan and protein were relatively high when grown in clay. Protein and starch contents of the grain kernel are important from a nutritional point of view and they are the main components determining baking quality. The damaged starch content correlated positively with total starch content and correlated inversely with protein content (Supplementary Table S2). Starch damage affects technological properties such as water absorption, which affects dough mixing properties of the flour.

β-Glucan is one of the health-promoting compounds in oats with approved health claims. When β-glucan content is an important trait from a selling point of view in relation to the maintenance and improvement of health, varieties can be selected that give high yield on clay soil in combination with high β-glucan concentration. This could also be combined with higher protein contents, antioxidant activity, and α-tocotrienol. High fiber content is desirable when oats are consumed as whole grain in porridge and as breakfast cereal. However, high β-glucan content has a negative effect on baking quality by increasing water absorption and decreasing dough plasticity [37,38,39].

**Figure 2:**
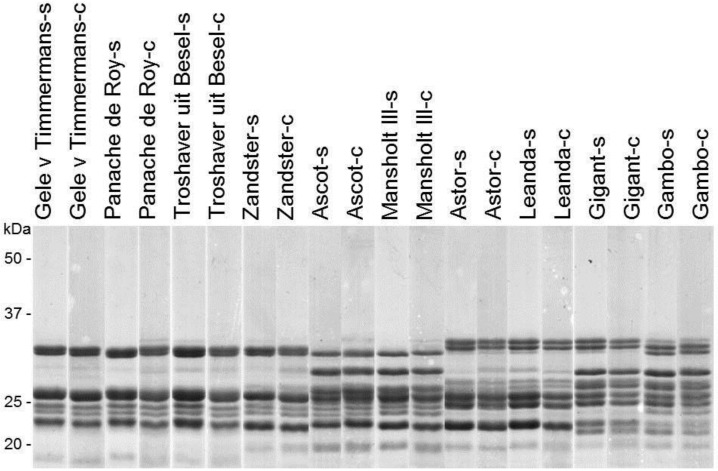
Avenin extracts (2 μg) from ten oat varieties grown in sandy (s) and clay (c) soil separated by SDS-PAGE gels and stained with PageBlue™. Lanes were obtained from three SDS-PAGE gels run at the same time.

Avenins are storage proteins from oats comprising about 10% to 13% of the total protein content. The main storage proteins in oats are the globulins that comprise about 55% of the total protein. Proteins in oats have an overall higher lysine content and a lower glutamine and proline content compared with other cereals [40]. Therefore, the proteins fit well to human and animal needs of amino acids in food and feed. The avenin contents of the oat varieties used in this study were also higher when grown in clay soil compared with sandy soil and correlated to the total protein content (Table 1 and Table S1). Cultivated oats are hexaploid and contain 7–12 storage proteins called avenins as analyzed by SDS-PAGE (Figure 2). Figure 2 shows the number of proteins present per variety and the similarity between varieties. Prolamins from oats may be involved in the technological properties in relation to baking quality. These prolamins have also been described as lacking the celiac disease-stimulating epitopes in contrast to prolamins from wheat, rye, and barley [4].

Avenin protein expression patterns showed some differences among varieties but no differences in avenin protein patterns were observed for the varieties when grown in either clay or sandy soil. Some of the varieties could be grouped together based on avenin patterns and this grouping was the same as for the groups obtained with PCA performed for the other compounds. At this moment it is unknown how avenin composition is related to crop properties. Therefore the data of the protein patterns could not be incorporated and combined with the other obtained results.

The fatty acid levels were affected by variety and soil, where sandy soil mostly resulted in higher levels except for polyunsaturated fatty acid (PUFA) C18:3 (linolenic acid). Since C18:3 covered only 3% of the total PUFAs in oats, this could be neglected. In contrast, C18:2 (linoleic acid) covered 97% of the PUFAs. It also covered >40% of the total fatty acid content in all varieties. Both C16:0 and C18:0 contents were main contributors of the total content of saturated fatty acids (SFAs) of which the contribution of C16:0 was the highest (>90%). The ratio of UFAs (MUFAs + PUFAs) to SFAs ranged from 4.0–4.6 to 1, ratios of PUFAs to SFAs ranged from 2.3–2.6 to 1. Results show that the concentrations of different types of fatty acids are affected differently. This might be caused by their presence in different tissue types of the oat grain. Banaś *et al.* [41] showed that the embryo had higher levels of C18:2 and C18:3 PUFAs compared with whole grain, levels of C18:1 were higher in whole grain, and the embryo plus scutellum had a higher level of C16:0. The main part of the fatty acids was present in the endosperm.

The two different forms of vitamin E in oats, α-tocopherol and α-tocotrienol, were also differently affected. α-Tocopherol is located mostly in the germ and α-tocotrienol is located mostly in the endosperm [42,43]. α-Tocotrienol has been reported to be a strong antioxidant, and high intake of vitamin E from food may contribute to the prevention of age-dependent neurodegenerative diseases such as Alzheimer’s Disease [22] Vitamin E refers to a group of eight fat-soluble compounds that include both tocopherols (α-,β-, γ-, and δ-tocopherols) and tocotrienols (α-,β-, γ-, and δ-tocotrienols) [44]. Cereal grains, such as oats, and some vegetable oils are the major food sources for tocotrienols in the Western diet [22,45]. Total tocol content can be affected by storage and temperature, and tocol concentrations seem to decrease when stored at room temperature, especially when exposed to air, but are stable when frozen (<−20 °C). Vitamin E is also subject to oxidation by lipoxygenase, which needs to be inactivated by hydrothermal processes [46], however, these thermal processes also affect tocol concentrations. Autoclaving of oat grains for 16 min at 100–120 °C increased α-tocopherol and α-tocotrienol contents, while steaming (1 h at 100 °C) and flaking decreased α-tocotrienol content but not α-tocopherol content [47]. For kilning we used a rather short steaming step (3 min at 100 °C) to stop oxidation by lipoxygenase and lipase and also to prevent rancidity, however, this may have altered the tocol contents. The choice of treatment to inactivate oxidation and prevent rancidity is therefore an important first step before oat grain kernels are further processed. Most varieties showed higher α-tocotrienol content when grown in clay soil. No significant differences were observed in α-tocopherol content when grown in clay or sandy soil for all varieties, but differences were observed among varieties (Table 1 and Table S1).

Antioxidant activity is another important health-related characteristic. Antioxidant activity in oats is mainly caused by the presence of phenolic compounds and vitamins. The majority of phenolic compounds in oats are avenanthramides [48] with three major forms A, B, and C, that are rather heat stable [49,50]. The A form is somewhat reduced by steaming and flaking of de-hulled oats, whereas the B and C forms are not [47]; therefore, the antioxidant activity measurements reported here probably mostly concern the B and C forms. Antioxidant activities were different among varieties, although all levels were higher when grown in clay soil (Table 1 and Table S1).

Values of agronomical importance for oats are yield, TKW and resistance to lodging. Yields were different among varieties, and some varieties gave higher yields when grown in clay, while other varieties gave higher yields when grown in sand. Values for yield and TKW correlated positively. They both correlated negatively with protein content and positively with starch content (Table S2).

Resistance to lodging is another important agronomical characteristic for growing oats. When varieties are not resistant to lodging, this can have an enormous effect on the yield. The stalks of the plants start to bend and finally fall on the ground. In our study, most oat varieties were very resistant to lodging when grown in sandy soil and some varieties showed also high resistance to lodging when grown in clay. Yields tended to be higher when grown in clay (Table 1 and Table S1). TKW was similar when grown in clay and sand indicating that the different compounds compensate each other in the total kernel weight.

## 4. Conclusions

The results show the variation among oat genotypes and the extent of the responses of several important metabolites and agricultural characteristics of oat varieties to the major environmental condition, that is, the soil type. The variation means that a particular oat variety will not always produce the same quality for industrial purposes, as is known for other cereal species, but also contains different levels of health-related compounds. This knowledge is relevant in oat breeding and for selection of varieties when aiming at specific compound/metabolite profiles for specific food, feed or industrial applications and innovations.

## Supplementary Files

Supplementary File 1
