# Supplementary material for: Profiling of Nutritional and Health-Related Compounds in Oat Varieties
Source: Foods. 2015 Dec 25;5(1):2. doi: 10.3390/foods5010002 (PMC5224580; doi:10.3390/foods5010002)
Supplement: Supplementary File 1 [file foods-05-00002-s001.pdf]

Supplementary Materials

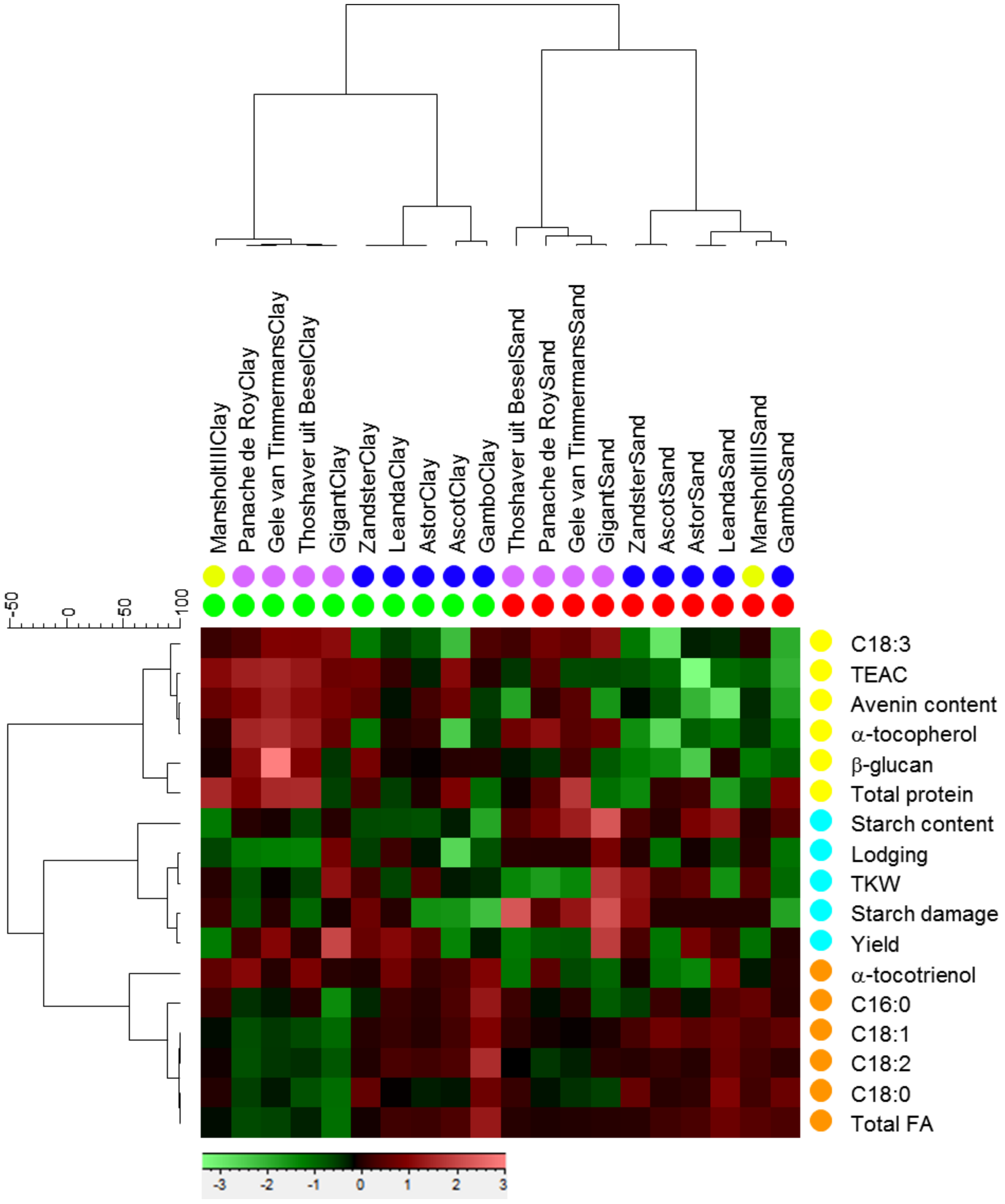

Figure S1. Cluster analysis showing groups identified for oat varieties (top) and compounds (right) in different colored dots.

Table S1. Values for health-related, nutritional compounds and field characteristics per oat variety grown in sand and clay soil.

Variety	Soil	Total Protein	Avenins	Starch	Starch Damage	β-Glucan	Fatty Acids (mg/100 g)					Total FA	TEAC	Vitamin E (mg/100g)		Yield	TKW	Lodging
		(%)	(%)	(%)	(%)	(%)	C16:0	C18:0	C18:1	C18:2	C18:3	%	(μmol/ 100 g)	α-Tocopherol	α-Tocotrienol	(kg/ha)	(g)	
**Ascot**	**Sand**	13.3	0.5	52.4	2.1	3.6	245.9	17.4	548.5	622.1	12.3	1.8	297.0	0.7	3.0	4940	31	6
	**Clay**	14.9	0.7	52.2	1.4	4.5	262.3	17.4	518.6	662.6	13.3	1.7	478.6	0.8	3.7	4993	30	4
**Astor**	**Sand**	13.9	0.4	59.7	2.3	3.3	241.6	19.6	549.6	636.3	15.4	1.7	190.6	1.2	3.0	7161	33	9
	**Clay**	13.7	0.7	50.5	1.5	4.5	257.0	18.1	508.1	666.1	15.0	1.6	373.6	1.6	3.7	6926	33	9
**Gambo**	**Sand**	16.6	0.5	63.5	1.8	4.6	291.9	33.3	718.1	730.9	14.7	2.0	312.1	1.4	4.1	7401	31	9
	**Clay**	13.0	0.7	47.2	1.4	4.9	331.1	29.9	723.3	849.0	17.1	1.9	442.2	1.6	4.4	6650	32	9
**Gele van Timmermans**	**Sand**	15.6	0.6	59.8	2.9	4.4	235.2	14.1	421.4	575.0	15.9	1.4	305.4	1.5	3.1	5071	25	8
	**Clay**	13.8	0.6	45.8	1.5	5.6	197.5	11.0	312.6	500.3	15.3	1.2	395.5	1.6	3.0	5893	26	4
**Gigant**	**Sand**	10.9	0.4	63.0	3.4	3.6	193.5	11.5	385.0	566.2	16.0	1.4	278.3	1.4	2.8	7302	34	9
	**Clay**	11.6	0.6	48.9	1.7	3.8	178.7	10.0	310.5	513.0	16.1	1.2	373.0	1.4	3.2	7570	32	9
**Leanda**	**Sand**	12.1	0.4	66.5	2.8	4.9	292.9	32.6	674.0	748.9	16.1	1.8	356.5	1.3	4.4	7246	28	9
	**Clay**	12.4	0.6	48.4	2.1	4.3	245.9	16.4	472.4	637.3	14.8	1.6	373.1	1.4	3.8	7077	28	9
**Mansholt III**	**Sand**	12.4	0.6	53.2	2.2	3.8	268.2	19.6	515.9	645.9	15.7	1.6	314.6	1.3	3.4	5119	32	9
	**Clay**	15.5	0.7	45.6	2.2	4.3	246.8	17.2	436.7	603.2	15.6	1.5	452.5	1.4	3.7	4880	30	7
**Panache de Roy**	**Sand**	12.9	0.5	53.2	2.0	3.8	213.2	13.1	385.2	532.3	15.6	1.3	351.7	1.6	3.4	4698	23	7
	**Clay**	13.8	0.7	49.5	1.4	4.7	210.7	12.2	343.4	524.1	15.3	1.2	440.6	1.8	3.7	5687	26	5
**Troshaver uit Besel**	**Sand**	12.7	0.4	53.1	4.0	4.1	239.4	16.8	444.1	583.2	15.5	1.4	316.7	1.6	2.9	4746	25	8
	**Clay**	15.0	0.7	46.8	1.4	4.7	228.9	12.1	358.8	555.7	16.2	1.3	442.6	1.8	3.7	5657	27	5
**Zandster**	**Sand**	11.5	0.6	55.5	3.1	3.8	228.7	22.6	512.5	627.9	14.3	1.5	326.7	1.0	3.5	6496	35	9
	**Clay**	12.8	0.6	45.6	2.2	4.5	211.0	17.5	400.9	568.3	13.5	1.4	381.0	0.9	3.2	6061	29	6

Table S2. Correlation coefficients obtained data.

	β-Glucan	C16:0	C18:0	C18:1	C18:2	C18:3	Total FA	α-Tocopherol	α-Tocotrienol	Starch	Starch Damage	Total Protein	Avenin Content (%)	Yield	TKW	Lodging
TEAC	0.643	0.100	−0.100	0.075	0.021	0.047	−0.063	0.216	0.499	−0.645	−0.576	0.234	0.759	−0.252	−0.259	−0.550
β-glucan		0.240	0.182	0.048	0.149	0.150	0.117	0.371	0.483	−0.300	−0.447	0.369	0.458	0.004	−0.400	−0.450
C16:0			0.858	0.919	0.949	0.101	0.957	−0.045	0.729	0.226	−0.124	0.286	0.014	0.073	0.187	0.315
C18:0				0.946	0.894	0.029	0.934	−0.183	0.699	0.454	0.049	0.133	−0.218	0.340	0.301	0.451
C18:1					0.954	−0.040	0.989	−0.254	0.629	0.454	0.007	0.155	−0.215	0.286	0.378	0.459
C18:2						0.095	0.986	−0.160	0.720	0.299	−0.075	0.092	−0.073	0.313	0.401	0.456
C18:3							0.034	0.765	0.233	0.100	0.066	−0.105	−0.098	0.246	−0.125	0.393
Total FA								−0.189	0.690	0.375	−0.039	0.154	−0.136	0.272	0.362	0.447
α-tocopherol									0.192	−0.121	−0.156	0.152	0.109	0.028	−0.466	0.047
α-tocotrienol										0.050	−0.418	0.193	0.286	0.290	0.062	0.172
Starch											0.513	−0.047	−0.667	0.339	0.172	0.492
Starch damage												−0.393	−0.720	−0.058	0.008	0.432
Total protein													0.453	−0.300	−0.279	−0.369
Avenin content														−0.252	−0.053	−0.472
Yield															0.542	0.569
TKW																0.480
